# Emerging therapies for HBsAg seroclearance: spotlight on novel combination strategies

**DOI:** 10.1007/s12072-025-10828-0

**Published:** 2025-06-11

**Authors:** Rex Wan-Hin Hui, James Fung, Wai-Kay Seto, Man-Fung Yuen, Lung-Yi Mak

**Affiliations:** 1 Department of Medicine, Queen Mary Hospital, The University of Hong Kong, Pokfulam Road, Pok Fu Lam, Hong Kong; 2 State Key Laboratory of Liver Research, The University of Hong Kong, Pok Fu Lam, Hong Kong

**Keywords:** HBsAg, HBV, HCC, Combination, siRNA, ASO, Immunomodulators, Immune checkpoint inhibitors, T cells, Vaccine

## Abstract

**Introduction:**

Functional cure is a favorable endpoint in chronic hepatitis B (CHB), yet it is rarely achieved with currently approved drugs (nucleos[t]ide analogues and pegylated interferon alpha). A range of novel agents, broadly classified into virus-targeting agents and immunomodulators, are hence developed with functional cure as the treatment target. As the data on individual novel agents are maturing, the field has gradually shifted to novel combination strategies.

**Methods:**

This article comprehensively reviewed the data on novel combination strategies against CHB. Potential mechanisms and future developmental directions are also discussed

**Results:**

RNA silencers (including antisense oligonucleotides and small-interfering RNAs) form the backbone of most combination strategies. Synergistic effects are observable with the combination of RNA silencers + single or dual immunomodulators, primarily through enhancing the magnitude and rate of hepatitis B surface antigen (HBsAg) decline, prolonging RNA silencer effects, and reducing HBsAg rebound after end-of-treatment. Accumulating data also demonstrate immune dysfunction recovery among patients with significant HBsAg reduction on RNA silencer-based or immune checkpoint inhibitor-based combination therapies.

**Conclusion:**

Functional cure is now an attainable endpoint with novel combination treatment. Research is warranted to optimize combination regimens, and personalization of treatment strategies will be necessary. With further development, novel combination strategies have the potential to transform future CHB management.

## Introduction

Chronic hepatitis B (CHB) is one of the most prevalent chronic liver diseases globally. In 2024, 3.0% of the global population was infected by the hepatitis B virus (HBV), with HBV accounting for over 900,000 new hepatocellular carcinoma (HCC) cases and 1.1-million liver-related mortalities [1]. The global health burden of CHB is perpetuated by the unique viral features of covalently-closed circular DNA (cccDNA) and DNA integration [2], both of which preclude the complete and sterilizing cure of HBV. Instead of complete cure, an alternate endpoint that is currently achievable is functional cure, defined as sustained hepatitis B surface antigen (HBsAg) seroclearance with unquantifiable HBV DNA at 24 weeks off treatment [3].

Functional cure is associated with liver fibrosis regression [4, 5] and reduced HCC risks [6]. Furthermore, functional cure represents restored host immunity against HBV, and is considered as a quiescent disease phase [7]. Despite the allure of functional cure, it is a rarely achieved endpoint, and cannot be consistently induced by the currently approved HBV drugs of nucleos(t)ide analogs (NUCs) and pegylated interferon alpha (Peg-IFNα). NUCs, as the main HBV treatment option worldwide, can potently suppress HBV replication [7], but has minimal effects on HBsAg [8, 9]. In a large meta-analysis involving 34 studies and 42,588 patients, the annual HBsAg seroclearance rates were statistically comparable between NUC-treated and untreated patients (0.8% and 1.3%, respectively) [10].

With the limitations of current HBV drugs, a range of novel agents targeting functional cure are emerging. These novel agents can be broadly classified into virus-targeting agents—drugs that target distinct steps of the viral lifecycle, and immunomodulators—drugs that enhance host anti-HBV immune responses [2]. While the data on individual novel agents are maturing, newer trials have further established synergistic antiviral effects when combining these emerging drugs [11]. This article will review the latest evidence and discuss the role of emerging combination strategies for functional cure.

## Novel agents in development

### Virus-targeting agents

**Fig. 1:**
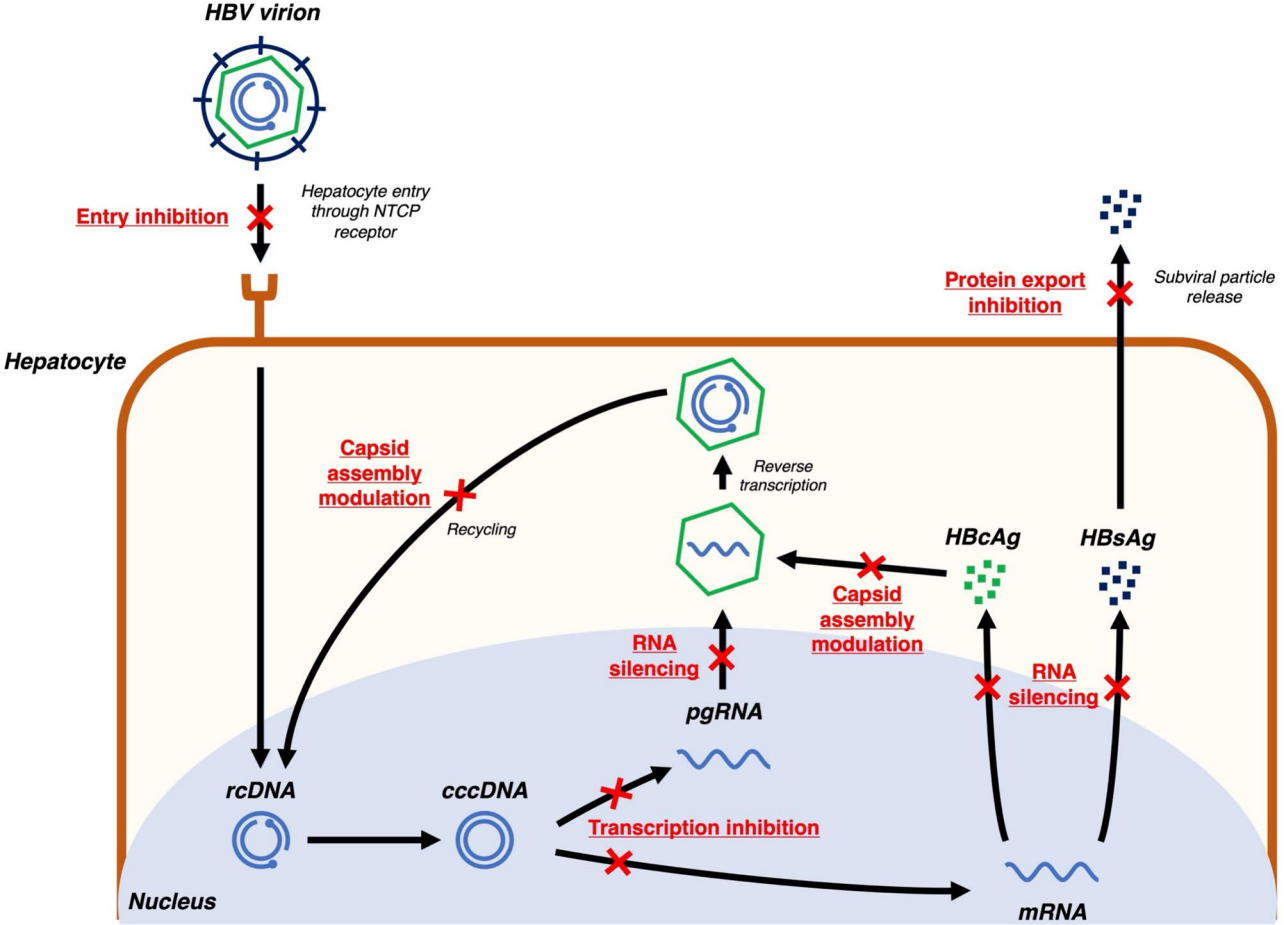
Novel virus-targeting agents against hepatitis B. *cccDNA* covalently closed circular DNA, *HBcAg* hepatitis B core antigen, *HBsAg* hepatitis B surface antigen, *HBV* hepatitis B virus, *mRNA* messenger RNA, *pgRNA* pregenomic RNA, *rcDNA* relaxed circular DNA

HBV is an enveloped partially double-stranded DNA virus with a relaxed circular DNA (rcDNA) genome of 3200 base pairs [12]. The HBV lifecycle has multiple druggable targets that have been studied for novel HBV therapies (Fig. 1).

First, since HBV interacts with sodium taurocholate cotransporting polypeptide (NTCP) receptors for cellular uptake [13], competitive entry inhibitors of the NTCP receptor have been developed. Bulevirtide is the first-in-class entry inhibitor, which has primarily been studied and granted conditional approval for therapeutic use in Europe in HBV and hepatitis D virus (HDV) coinfected patients, as HDV has limited therapeutic targets [14]. However, there is still a lack of studies to assess bulevirtide in HBV mono-infected populations.

After HBV enters hepatocytes, it is uncoated and the viral rcDNA is transported to the hepatocyte nuclei. Using host cell repair mechanisms, the rcDNA is ligated to form cccDNA, which acts as a transcription template for viral messenger RNA (mRNA) and pregenomic RNA (pgRNA) [15]. This transcription step can be inhibited by modulation of farnesoid X receptors (FXR), which in turn inhibit downstream viral replication. Selective FXR agonists such as vonafexor have been studied in phase II clinical trials [16].

HBV mRNAs are translated to multiple viral proteins including HBsAg, hepatitis B e antigen (HBeAg), hepatitis B core antigen (HBcAg), HBV X protein, and HBV polymerase [15]. RNA silencers, including small-interfering RNAs (siRNAs) and antisense oligonucleotides (ASOs), are designed with complementary nucleotide sequences to conserved regions of the HBV genome, enabling effective silencing of mRNA translation [17]. With the potent suppression of mRNA translation, RNA silencers reduce circulating viral protein levels, indirectly enhancing host immune reconstitution against HBV [18]. Furthermore, RNA silencers can inhibit downstream pgRNA activity, in turn inhibiting viral replication [17]. RNA silencers are the most advanced drug class in development when compared with other novel drug candidates. Multiple agents including bepirovirsen (ASO), daplusiran/tomligisiran (siRNA, also known as JNJ-3989), elebsiran (siRNA, also known as VIR-2218), imdusiran (siRNA, also known as AB-729), RBD-1016 (siRNA), and xalnesiran (siRNA, also known as RO7445482) have entered phase II or III trials [19]. Details on therapeutic targets and treatment efficacy of ASOs and siRNAs are described in Table 1 [20–25].

**Table 1:** Targets and treatment efficacy of RNA silencers

Drug class	Agent	Therapeutic target	Treatment efficacy in monotherapy trials
ASO	Bepirovirsen	Targeting a highly conserved region in the HBV genome, enabling targeting of all HBV RNA (including pgRNA and mRNA)	Primary outcome (undetectable HBsAg and unquantifiable HBV DNA at 24 weeks after EOT) occurred in 5.7% (26/457) of patients In patients with baseline HBsAg ≤ 3 log IU/ml who were given bepirovirsen 300 mg weekly for 24 weeks with loading dose, the primary outcome occurred in 25% of non-NUC patients and 16% of NUC treated patients
siRNA	Daplusiran/tomligisiran	Dual triggers targeting the HBV X and S ORFs	Dose-dependent HBsAg suppression was noted Among patients receiving daplusiran/tomligisiran at doses between 100–400 mg, 97.5% achieved ≥ 1 log IU/ml reduction in HBsAg, and 75.0% of patients achieved HBsAg < 100 IU/ml at EOT
	Elebsiran	Targeting the HBV X ORF	Among patients on elebsiran 200 mg, maximum HBsAg reduction by 1.65 log IU/ml was noted 50% of patients on elebsiran achieved HBsAg < 100 IU/ml
	Imdusiran	Targeting the HBV X ORF	HBsAg suppression was similar regardless of imdusiran dosing (60 mg or 90 mg) EOT HBsAg reduction by 1.8–2.6 log IU/ml was achieved
	RBD-1016	Targeting the HBV X ORF	Multiple dosing day 1 and day 29) of RBD-1016 at 3.0 mg/kg (led to HBsAg reduction by 1.26 log IU/ml
	Xalnesiran	Targeting the HBV S ORF	Xalnesiran at 3.0 mg/kg and 6.0 mg/kg led to EOT HBsAg reduction by 1.91 and 1.87 log IU/ml respectively 91.7% of patients achieved HBsAg reduction by > 1 log IU/ml, and 58.3% achieved HBsAg < 100 IU/ml

*ASO* antisense oligonucleotide, *EOT* end-of-treatment, *HBsAg* hepatitis B surface antigen, *HBV* hepatitis B virus, *mRNA* messenger RNA, *ORF* open reading frame, *pgRNA* pregenomic RNA, *siRNA* small interfering RNA

HBcAg is one of the viral proteins translated from HBV mRNA, and is an important component of HBV nucleocapsids [26]. Capsid assembly modulators (CAMs) interfere with HBcAg to induce formation of aberrant nucleocapsids (CAM-A action) or empty nucleocapsids (CAM-E action)—constituting their primary action [27]. CAMs also have a secondary mode of action in stimulating mistimed uncoating of nucleocapsids, which interferes with cccDNA recycling [28]. ALG-000184 is a newer generation CAM with both the strongest primary and secondary modes of action among all developing CAMs, and its development has entered phase II [29].

HBsAg, aside from being the key component of the viral envelope, is also released into the circulation as subviral particles [15]. Nucleic acid polymers (NAPs) such as REP2139 and REP2165 are effective in blocking subviral particle export from hepatocytes, in turn reducing HBsAg levels [30].

### Immunomodulators

**Fig. 2:**
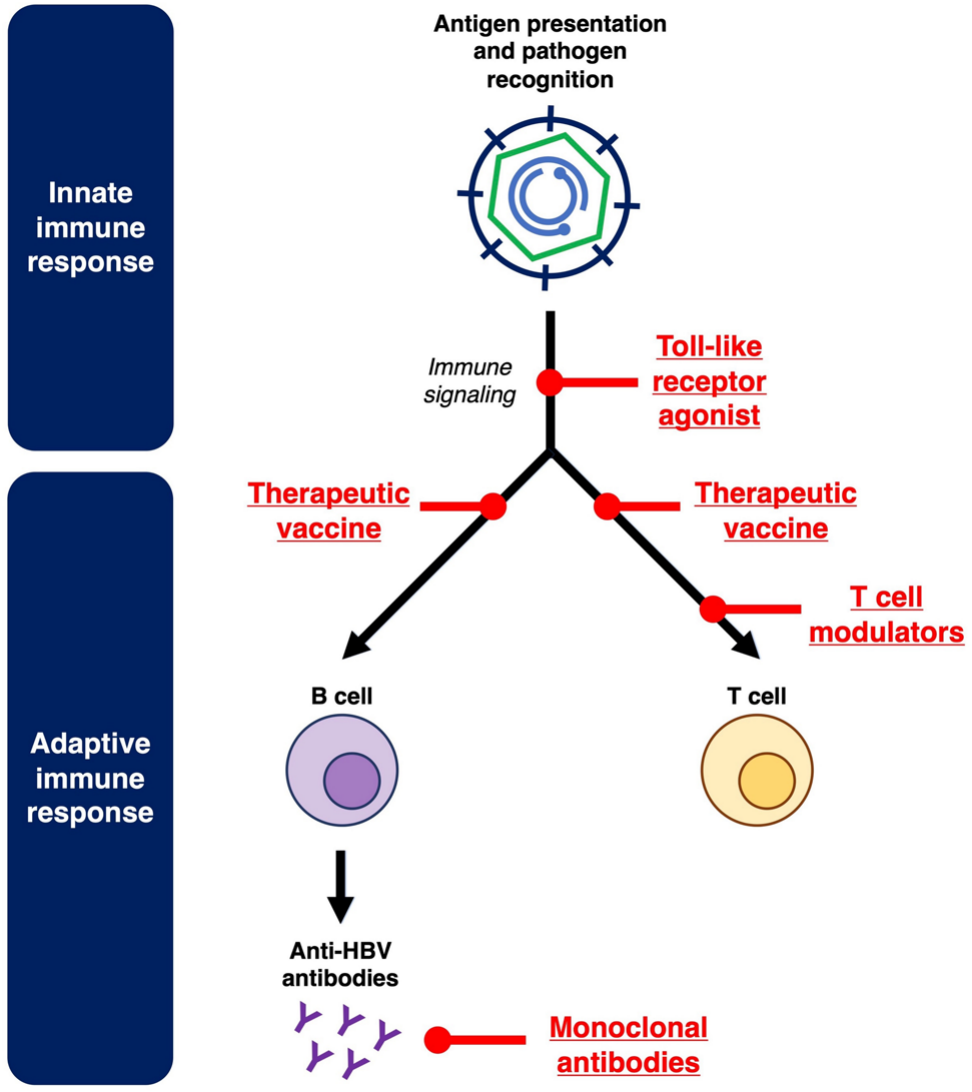
Novel immunomodulators against hepatitis B

Host immunotolerance is a well-established pathogenic mechanism contributing to CHB. Both innate and adaptive immune responses can be influenced by prolonged exposure to HBsAg and other viral proteins, ultimately resulting in immune dysfunction and chronic HBV persistence [31, 32]. Immunomodulators target the host immune system to reverse immunotolerance and stimulate anti-HBV immune responses. The key immunomodulatory pathways targeted in novel CHB treatment are depicted in Fig. 2.

First, toll-like receptors (TLRs) are critical for pathogen recognition in the innate immune system [33]. TLR7 and TLR8 agonists, which can stimulate downstream cytokine release and immune signaling, have both been studied as novel agents against CHB [34, 35].

Therapeutic vaccination has been studied as a strategy to generate active immunity against HBV. While older therapeutic vaccines adopted HBsAg as the main constituent [36], newer data supports the incorporation of other viral proteins. These newer generation therapeutic vaccines such as BRII-179 (consisting of 3 HBV surface antigens PreS1, PreS2 and S) [37] and VTP-300 (consisting of polymerase, core, entire S region) [38] have entered phase II trials. In contrast to active immunity through vaccination, passive immunity through administration of anti-HBV monoclonal antibodies is another strategy that has been studied. In addition, these monoclonal antibodies exert some vaccinal effects [39, 40]. With the emergence of mRNA vaccine technology, therapeutic HBV vaccines on mRNA platforms have also been developed. Nonetheless, these mRNA vaccines remain in in-vitro and animal studies, and have yet to be studied in clinical trials [41–43].

Finally, immunomodulators against T cells have been studied to reverse T cell dysfunction in CHB [32]. Immune checkpoint inhibitors against the Programmed cell death protein 1/Programmed cell death ligand 1 (PD1/PDL1) pathway have entered clinical trials for CHB [44, 45]. Novel techniques such as Inhibitors of apoptosis (IAP) antagonists and immune mobilizing monoclonal T cell receptors against virus (ImmTAV) are other T cell modulators that have been studied [46, 47].

### An overview on the developmental landscape for novel agents

The treatment efficacy from novel agent monotherapies is summarized in Tables 1 and 2 [48]. Currently, RNA silencers are the most promising novel agents in development, demonstrating potent and sustainable HBsAg suppression [49]. HBsAg seroclearance has also been documented with RNA silencer therapy, although this is mostly seen in patients with lower baseline HBsAg (< 1000 IU/ml) [20]. RNA silencers will likely form the backbone for future HBV therapy. CAMs are another drug class with encouraging trial results. ALG-000184, a newer generation CAMs, can induce deeper HBV DNA suppression than NUCs alone, and has also shown HBsAg reduction effects [29]. In contrast to virus-targeting agents, immunomodulators are generally less potent in HBsAg suppression, and have limited roles in monotherapy.

**Table 2:** Overview on novel agents outside of RNA silencers

	Class	Agents	Overview on monotherapy trial findings
Virus-targeting agents	Entry inhibitor–NTCP receptor antagonist	Bulevirtide	Primarily studied in HBV-HDV co-infected patients Minimal effects on HBsAg seroclearance or reduction
	Transcription inhibitor–FXR agonist	Vonafexor	Minimal effects on HBsAg reduction (0.1 log IU/ml reduction after 29 days of treatment) No effects on other HBV markers
	CAMs	CAM-A GLS4 RO7049389 CAM-E ALG-000184 Bersacapavir	Earlier generation CAMs had limited effects on HBsAg and other viral proteins Newer generation CAMs (e.g., ALG-000184) have increased potency. As monotherapy, ALG-000184 can induce deeper HBV DNA suppression than NUCs In treatment up to 96 weeks in ongoing trials, ALG-000184 can induce > 1 log IU/ml reduction in HBsAg, HBeAg and HBcrAg
	Protein export inhibitor–NAPs	REP2139/REP2165	48 weeks of treatment led to 60% (24/40) of patients having HBsAg suppressed to below 0.05 IU/ml. This effect was sustainable in 35% of patients (14/40) at 48 weeks after EOT
Immunomodulators	Toll-like receptor agonist	Toll-like receptor 8 agonist Selgantolimod Toll-like receptor 7 agonist Ruzotolimod Vesatolimod	When administered in NUC treated patients, selgantolimod induced HBsAg seroclearance in 5% of patients (2/39) at 24 weeks after EOT Toll-like receptor 7 agonist had minimal effects on HBsAg
	T-cell modulators	Anti PD1/PDL1 Envafolimab Cemiplimab Nivolumab RO7191863 (PD-L1 LNA) Inhibitors of apoptosis antagonist APG1387 Immune mobilizing monoclonal T-cell receptors against virus IMC-I109 V	Effects of anti PD1/PDL1 most prominent in patients with lower baseline qHBsAg. With 24 weeks of envafolimab, EOT HBsAg reduction was 1.38 log IU/ml, 0.19 log IU/ml, and 0.09 log IU/ml in patients with baseline qHBsAg ≤ 100 IU/ml, 100–1000 IU/ml, and > 1000 IU/ml respectively Outside of envafolimab, the other T-cell modulators are studied in ongoing clinical trials
	Therapeutic vaccines	VTP-300 BRII-179 GSK3528869 A	VTP-300 induced HBsAg decline by over 0.5 log IU/ml in 3 out of 18 patients. Yet these patients all had pretreatment qHBsAg < 50 IU/ml BRII-179 did not induce significant HBsAg reduction
	Anti-HBV Monoclonal antibodies	Lenvervimab Tobevibart	Tobevibart for 8 weeks induced HBsAg reduction by 1.8 log IU/ml Lenvervimab for 4 weeks induced undetectable HBsAg in 3 out of 29 patients, although HBsAg levels rebounded after EOT

*CAM* capsid assembly modulator, *EOT* end-of-treatment, *FXR* farnesoid X receptor, *HBV* Hepatitis B virus, *HDV* hepatitis D virus, *LNA* locked nucleic acid, *NAP* nucleic acid polymer, *NTCP* sodium-taurocholate co-transporting polypeptide, *NUC* nucleos(t)ide analog, *PD1* programmed cell-death 1, *PDL1* programmed cell-death ligand 1, *SC* subcutaneous, *siRNA* small-interfering RNA

Based on the current trial results, no novel agents can consistently induce functional cure at high rates as monotherapy. While HBsAg reduction is a key study outcome in multiple clinical trials, HBsAg suppression alone does not guarantee subsequent HBsAg seroclearance. Host immune response boosting, either directly by immunomodulators or indirectly via viral antigen reduction, is also essential for HBsAg seroclearance. Interest in combination strategies of novel agents has hence grown, as combination treatment can target multiple pathways concurrently to yield synergistic antiviral effects. The following sections will provide an in depth review on combination strategies in clinical trials.

## Combination strategies in clinical trials

### Combination of virus-targeting agents

siRNAs and CAMs, as two of the most well-studied novel drug classes, have been studied in multiple combination trials (Table 3). The REEF-1 and REEF-2 trials both studied the combination of daplusiran/tomligisiran (siRNA) + bersacapavir (CAM).

**Table 3:** Combination strategies with novel agents

	Combination regimen	Study findings	Clinical trial phase
Combination of virus-targeting agents	Daplusiran/tomligisiran (siRNA) + bersacapavir (CAM)	Patient population in the REEF-1 trial: Patients with qHBsAg > 100 IU/ml, including both HBeAg positive/negative and NUC treated/untreated patients Patient population in the REEF-2 trial: NUC treated HBeAg negative patients with qHBsAg > 100 IU/ml Treatment efficacy were driven by daplusiran/tomligisiran Addition of bersacapavir did not lead to synergistic HBsAg suppression or HBsAg seroclearance	II
	Imdusiran (siRNA) + vebicorvir (CAM)	Patient population: NUC treated HBeAg negative patients with qHBsAg ≥ 100 IU/ml Addition of vebicorvir to imdusiran did not yield synergistic HBsAg suppression, and did not increase probability of reaching a predefined stop NUC criteria	II
Combination of immunomodulators	VTP-300 (therapeutic vaccine) + nivolumab (anti PD1)	Patient population in the phase Ib/IIa trial: NUC treated patients with qHBsAg < 4000 IU/ml Patient population in the phase IIb HBV003 trial: NUC treated patients with qHBsAg ≤ 200 IU/ml EOT HBsAg seroclearance occurred in 11.1–11.6% of the best performing arms on VTP-300 + nivolumab Timing of nivolumab (with boost dose vaccine vs with boost + prime dose vaccines) influenced treatment effects	I/II
	BRII-179 (therapeutic vaccine) + Peg-IFNα	Patient population: NUC treated patients with qHBsAg between 50–5000 IU/ml BRII-179 + Peg-IFNα did not lead to significant HBsAg reduction	I/II
	TQ-B2450 (anti-PDL1) + TQ-A3334 (TLR7 agonist)	Patient population: NUC treated patients, including both HBeAg positive/negative patients EOT HBsAg reduction by 0.45 log IU/ml, which was higher than that in TQ-A3334 monotherapy Combination therapy also had greater suppression effects on HBV RNA and HBeAg when compared with TQ-A3334 monotherapy	II
	Nivolumab (anti PD1) + selgantolimod (TLR8 agonist)	Patient population: NUC treated HBeAg negative patients Treatment led to sustained elevation of cytokines and chemokines, with robust increase in soluble PD1 Minimal effects on HBsAg (EOT HBsAg reduction by 0.03 log IU/ml)	I
	GS-4224 (anti PDL1) + selgantolimod (TLR8 agonist)	Patient population: NUC treated HBeAg negative patients Minimal effects on inflammatory markers or on HBsAg (EOT HBsAg reduction by 0.02 log IU/ml)	I
Combination of RNA silencers with Peg-IFNα	Bepirovirsen (ASO) + Peg-IFNα	Patient population: NUC treated patients with qHBsAg > 100 IU/ml HBsAg seroclearance at 24 weeks post-EOT in 9–15% of patients HBsAg seroclearance only observed in patients with baseline HBsAg < 3000 IU/ml Peg-IFNα had no effect on maximal HBsAg suppression, but reduced HBsAg rebound after EOT	II
	Xalnesiran (siRNA) + Peg-IFNα or ruzotolimod (TLR7 agoinst)	Patient population: NUC treated patients HBsAg seroclearance at 24 weeks post-EOT occurred in 23% of patients on xalnesiran + Peg-IFNα vs 12% in xalnesiran + ruzotolimod HBsAg seroclearance only occurred in patients with baseline HBsAg < 3 log IU/ml	II
	Elebsiran (siRNA) + Peg-IFNα	Patient population: NUC treated patients with qHBsAg > 50 IU/ml Up to 29.7% of patients in the best performing treatment arm achieved EOT HBsAg seroclearance HBsAg seroclearance only occurred in patients with baseline HBsAg < 1500 IU/ml	II
	Imdusiran (siRNA) + Peg-IFNα	Patient population: NUC treated HBeAg negative patients 28% of patients achieved EOT HBsAg seroclearance Stopping NUC is feasible after combination therapy	II
	Daplusiran/tomligisiran + Peg-IFNα	Patient population: Untreated HBeAg positive patients with HBV DNA ≥ 20,000 IU/ml 20.3% of patients achieved HBsAg seroclearance at one or more timepoints in the trial Addition of Peg-IFNα accelerated HBsAg decline	II
Combination of RNA silencers with therapeutic vaccines	Imdusiran (siRNA) + VTP-300 (therapeutic vaccine) ± nivolumab (anti PD1)	Patient population: NUC treated patients EOT HBsAg < 100 IU/ml was achieved in 94.7% of patients on imdusiran + VTP-300, and in 92.3% of patients on imdusiran + VTP-300 + nivolumab 23.1% of patients on imdusiran + VTP-300 + nivolumab achieved undetectable EOT HBsAg Adding VTP-300 to imdusiran led to more sustained HBsAg suppression	II
	Daplusiran/tomligisiran (siRNA) + JNJ-0535 (therapeutic vaccine)	Patient population: NUC treated HBeAg negative patients Mean EOT HBsAg decline by 2.18 log IU/ml	I
	Elebsiran (siRNA) + BRII-179 (therapeutic vaccine)	Patient population: NUC treated patients Mean EOT HBsAg decline by 1.7 log IU/ml	II
Combination of RNA silencers with T cell modulators	Xalnesiran (siRNA) + RO7191863 (PD-L1 locked nucleic acid)	Patient population: NUC treated patients EOT HBsAg seroclearance occurred in 12.9% of patients on sequential combination treatment, and in 6.1% on concurrent combination treatment 6.5% of patients on sequential combination treatment achieved HBsAg seroclearance at 24 weeks post-EOT	II
	Daplusiran/tomligisiran (siRNA) + nivolumab (anti PD1)	Patient population: NUC treated HBeAg negative patients with qHBsAg ≤ 10,000 IU/ml EOT HBsAg reduction by 2.01–2.10 log IU/ml The HBsAg levels remained at 1.70–1.85 log IU/ml below baseline at 24 weeks post-EOT	II
	Elebsiran (siRNA) + nivolumab (anti PD1) + selgantolimod (TLR8 agonist)	Patient population: Patients with qHBsAg > 1.5 log IU/ml, including both NUC treated/untreated patients The triple regimen led to HBsAg seroclearance in 2.5% of non-NUC treated patients at 24 weeks post-EOT	II
Combination of RNA silencers with monoclonal antibodies	Elebsiran (siRNA) + tobevibart (monoclonal antibodies) ± Peg-IFNα	Patient population: NUC treated patients Among patients with baseline HBsAg < 1000 IU/ml, EOT HBsAg seroclearance was achieved in 45.5% on elebsiran + tobevibart + Peg-IFNα and in 38.9% on elebsiran + tobevibart The triple combination regimen enhanced sustainability of HBsAg suppression after EOT	II
Other combinations	Ledipasvir/sofosbuvir (NS5 A and NS5B inhibitor for HCV) + nivolumab (anti PD1)	Patient population: NUC treated HBeAg negative patients EOT HBsAg decline by 0.44 log IU/ml, although HBsAg rebounded to baseline at 24 weeks post-EOT	I

*ASO* antisense oligonucleotide, *CAM* capsid assembly modulator, *CHB* chronic hepatitis B, *EOT* end-of-treatment, *HBeAg* hepatitis B e antigen, *HCV* hepatitis C virus, *NUC* nucleos(t)ide analog, *PD-1* programmed cell-death 1, *Peg-IFNα* pegylated interferon alpha, *siRNA* small-interfering RNA

In the REEF-1 trial, patients were randomized to daplusiran/tomligisiran 100 mg + bersacapavir 250 mg, daplusiran/tomligisiran monotherapy (200 mg, 100 mg, or 40 mg arms), or bersacapavir 250 mg monotherapy for 48 weeks. The primary endpoint of the trial was a predetermined criteria for NUC withdrawal—HBsAg < 10 IU/ml, unquantifiable HBV DNA, and ALT < 3 × upper limit normal). Treatment efficacy appeared to depend on daplusiran/tomligisiran dosing. The daplusiran/tomligisiran 200 mg monotherapy group achieved the best results with end-of-treatment (EOT) HBsAg decline of 2.6 log IU/ml, with 19% of patients achieving the primary endpoint. In comparison, the daplusiran/tomligisiran 100 mg + bersacapavir 250 mg combination group achieved EOT HBsAg decline by 1.8 log IU/ml, with 9% achieving the primary endpoint. In the daplusiran/tomligisiran 100 mg monotherapy group, EOT HBsAg decline was 2.1 log IU/ml, with 16% achieving the primary endpoint. Three daplusiran/tomligisiran monotherapy patients (2 in the 200 mg arm and 1 in the 100 mg arm) achieved HBsAg seroclearance at 24 weeks post-EOT, whereas no patients on combination therapy achieved HBsAg seroclearance [50].

The REEF-2 trial studied daplusiran/tomligisiran 200 mg + bersacapavir 250 mg in NUC-treated patients, whereas the control arm received placebo. All treatment including NUCs were stopped after 48 weeks. 46.9% on combination treatment achieved HBsAg < 100 IU/ml at 48 weeks post-EOT, while only 15.0% of control patients achieved HBsAg < 100 IU/ml. No patients in the active or control arms achieved HBsAg seroclearance. As secondary endpoints, combination treatment reduced biochemical flares (3.6% vs 28.6% in controls) and also reduced the necessity to resume NUCs (9.1% vs 26.8% in controls) [51].

In the phase II trial of imdusiran (siRNA) + vebicorvir (CAM), combination treatment was compared against imdusiran monotherapy and vebicorvir monotherapy, respectively. After 48 weeks of treatment, 61.5% of patients on combination therapy achieved a predefined criteria to stop NUCs (HBsAg < 100 IU/ml, unquantifiable HBV DNA, and ALT < 2 × upper limit normal). In contrast, this stop NUC criteria was achieved in 80.0% of patients on imdusiran monotherapy and in 0% of patients on vebicorvir monotherapy [52].

According to the above findings, HBsAg reduction with the combination of siRNAs + CAMs was primarily driven by siRNA, and the addition of CAMs to siRNAs did not yield synergistic therapeutic effects.

### Combination of immunomodulators

Monotherapy with immunomodulators generally result in modest effects on HBsAg. Nonetheless, combining immune checkpoint inhibitors or therapeutic vaccines with other immunomodulators may concurrently boost the abundance and function of immune cells, in turn augmenting HBsAg suppressive effects.

VTP-300 is a therapeutic vaccine targeting HBsAg, HBcAg and HBV polymerase. It is a prime-boost vaccine, with the prime dose given at baseline and the boost dose given on day 28 [38]. A phase II trial studied different dosing regimens of nivolumab (anti PD1) in combination with VTP-300. The trial included patients receiving VTP-300 monotherapy, and also involved two combination arms with VTP-300 + nivolumab. Among the combination arms, one arm in the trial received a single dose of nivolumab with the boost dose vaccine (day 28), and the other arm received two doses of nivolumab with the prime (day 0) and boost (day 28) doses, respectively. The treatment efficacy was superior in patients who received VTP-300 + a single dose of day 28 nivolumab, as the group achieved mean HBsAg reduction of 0.76 log IU/ml at 2 months post-EOT, with 11.1% (2 out of 18 patients) attaining HBsAg seroclearance. The 2 patients who achieved HBsAg seroclearance had baseline HBsAg level of 61 IU/ml and 43 IU/ml respectively. Conversely, no significant HBsAg reduction or HBsAg seroclearance was achieved in patients who received VTP-300 with 2 nivolumab doses [38].

The ongoing HBV003 trial is studying VTP-300 + nivolumab in patients with baseline HBsAg < 200 IU/ml. At interim analysis, 11.6% of patients (8 out of 69) achieved HBsAg seroclearance, with 2 patients achieving anti-HBs seroconversion. Among 9 patients who discontinued NUCs at week 24 of the trial, 2 patients remained HBsAg negative, reaching the criteria for functional cure [53].

The trials on VTP-300 and nivolumab highlight the intricate interactions between different immunomodulators. While nivolumab can be synergistic with therapeutic vaccines, administration of nivolumab at specific timings may interfere with T cell activity to negate vaccinal effects.

BRII-179, another therapeutic vaccine, has been studied in combination with Peg-IFNα. This combination regimen successfully induced both T cell and antibody responses, yet it did not lead to significant HBsAg reduction [37].

The OCEANcure05 study is a phase II RCT studying the combination of TQ-B2450 (anti PDL1 monoclonal antibody) + TQ-A3334 (selective TLR7 agonist). This combination + NUC for 24 weeks led to mean EOT HBsAg reduction by 0.45 log IU/ml. In contrast, the EOT HBsAg reduction was 0.03 log IU/ml in the TQ-A3334 + NUC group, and 0.04 log IU/ml in the NUC only control group. The combination therapy also led to higher HBV RNA and HBeAg reduction compared to the other arms [54].

A recent open-label trial studied different combinations of immunomodulators, including nivolumab monotherapy, nivolumab + selgantolimod (TLR8 agonist), and GS4224 (a novel small-molecular PDL1 inhibitor) + selgantolimod. As HBsAg suppression was previously observed in HBV and hepatitis C virus (HCV) co-infected patients treated with ledipasvir/sofosbuvir, this trial also included a treatment arm with nivolumab + ledipasvir/sofosbuvir. Patients on nivolumab + ledipasvir/sofosbuvir had modest HBsAg decline by 0.44 log IU/ml at EOT, which rebounded to baseline after EOT. The other treatment arms did not have significant HBsAg decline during treatment. The primary endpoint (≥ 0.5 log IU/ml HBsAg decline from baseline to week 8 post-EOT) was only achieved in 1 patient (9%) in the nivolumab + ledipasvir/sofosbuvir arm and in 1 patient (8%) in the nivolumab + selgantolimod arm [55]. Sustained elevation of peripheral inflammatory cytokines and chemokines was observed in nivolumab monotherapy and in nivolumab + selgantolimod, suggesting target engagement [55]. This trial also demonstrated the potential effects of ledipasvir/sofosbuvir on HBsAg, although formal repurposing of this HCV drug for HBV treatment will require further validation.

### Combination of RNA silencers with Peg-IFNα

RNA silencers are the most potent virus-targeting agents in development [20, 49], and they have been the backbone in different combination strategies. The combination of RNA silencers + immunomodulators targets both the virus and the host, and should theoretically attain maximal therapeutic effects. RNA silencers can also induce immune reconstitution through reducing viral protein levels, in turn boosting the efficacy of immunomodulators. Among RNA silencer + immunomodulator combinations, multiple studies have been performed on Peg-IFNα—an approved drug for CHB.

Bepirovirsen (ASO) has been studied in combination with Peg-IFNα in NUC-treated patients in the B-Together trial. The primary outcome of the trial was undetectable HBsAg with unquantifiable HBV DNA at 24 weeks post-EOT. Among patients who received bepirovirsen 300-mg weekly (with loading doses) for 24 weeks followed by 24 weeks of Peg-IFNα, 9% (5 out of 55) achieved the primary outcome. Whereas among patients who received bepirovirsen 300 mg weekly (with loading doses) for 12 weeks followed by 24 weeks of Peg-IFNα, 15% (8 out of 53) achieved the primary outcome. All patients achieving the primary outcome had baseline HBsAg < 3000 IU/ml. When compared with data from the bepirovirsen monotherapy trial, Peg-IFNα reduced HBsAg rebound after EOT, yet had no effect on maximal HBsAg suppression [56].

A phase II trial studied the combination of xalnesiran + Peg-IFNα or ruzotolimod (TLR7 agonist) in NUC-treated patients. The primary endpoint of the study was HBsAg seroclearance at 24 weeks after EOT. In the xalnesiran + Peg-IFNα arm, patients simultaneously received xalnesiran 200 mg every 4 weeks + weekly Peg-IFNα for 48 weeks total. Whereas in the xalnesiran + ruzotolimod arm, patients received xalnesiran 200 mg every 4 weeks for 48 weeks + alternate day ruzotolimod 150 mg from weeks 13–24 and from weeks 37–48. The primary endpoint occurred in 23% of patients in the Peg-IFNα combination arm and in 12% in the ruzotolimod combination arm, which was superior to the effects in the xalnesiran monotherapy groups (7% in xalnesiran 100-mg group and 3% in xalnesiran 200 mg group). Baseline HBsAg was a key determinant of treatment effects, as HBsAg seroclearance only occurred in patients with baseline HBsAg < 3 log IU/ml. [57]

Elebsiran (siRNA) + Peg-IFNα has been studied in NUC-treated patients in phase II trials. Among 69 patients who received elebsiran (ranging from 6–13 doses) + Peg-IFNα (ranging from 12–48 doses), mean maximum HBsAg reduction by 1.7–3.0 log IU/ml was achieved. 15.9% of patients (11 out of 69) achieved undetectable HBsAg during the study, and only 6 patients had sustained HBsAg seroclearance at 24 weeks post-EOT. No patients receiving elebsiran monotherapy achieved HBsAg seroclearance [58].

Similar results have been demonstrated in another phase II trial on elebsiran (100 or 200 mg) + Peg-IFNα. In the combination therapy arms, 29.7% of patients (11 out of 37) achieved EOT HBsAg seroclearance. In contrast, only 5.6% (1 out of 18) on Peg-IFNα monotherapy achieved EOT HBsAg seroclearance. The addition of Peg-IFNα to elebsiran increased both the magnitude and rate of HBsAg suppression. All patients who achieved HBsAg seroclearance had baseline HBsAg < 1500 IU/ml [59].

The IM-PROVE I trial studied imdusiran (siRNA) + Peg-IFNα in HBeAg negative NUC-treated patients. Among 25 patients who received 24 weeks of Peg-IFNα, 28% of patients (7 out of 25) achieved undetectable HBsAg at EOT, with 6 of these 7 subjects having sustained HBsAg seroclearance at 24 weeks post-EOT. In contrast, no patients in the 12-week Peg-IFNα cohorts achieved HBsAg seroclearance. Overall, 21 patients met the stop NUC criteria (HBsAg < 100 IU/ml, undetectable HBV DNA, and ALT < 2 × upper limit normal) at 24 weeks after EOT, with only 5 patients requiring resumption of NUCs [60].

In contrast to other siRNA + Peg-IFNα trials which focused on NUC-treated patients, the REEF-IT trial (daplusiran/tomligisiran + Peg-IFNα) focused on immunotolerant patients with baseline HBV DNA > 4 log IU/ml. HBsAg seroclearance was achieved in 20.3% of patients (11 out of 54) at one or more timepoints in the trial, and 6 of these patients were still HBsAg negative at the last observed timepoint at interim analysis (up to 48 weeks post-EOT). Notably, the addition of Peg-IFNα primarily exerted its effect through accelerating HBsAg decline. [61]

### Combination of RNA silencers with therapeutic vaccines

The IM-PROVE II trial studied the combination of imdusiran + VTP-300 in NUC treated CHB patients. In the combination arm, 94.7% of patients (18 out of 19) achieved HBsAg < 100 IU/ml at EOT, while only 84.2% (16 out of 19) on imdusiran monotherapy achieved this outcome [62]. Overall, the addition of VTP-300 led to more sustained HBsAg suppressive effects from imdusiran. This trial had a further arm with addition of nivolumab on top of imdusiran + VTP-300. At EOT, 92.3% of patients (12 out of 13) on imdusiran + VTP-300 + nivolumab achieved HBsAg < 100 IU/ml, with 3 patients (23.1%) attaining undetectable EOT HBsAg [63].

The OSPREY trial is an ongoing phase Ib trial on the combination of daplusiran/tomligisiran + JNJ-0535 (therapeutic vaccine). At EOT, the combination treatment led to mean HBsAg decline by 2.18 log IU/ml. The combination treatment showed successful upregulation of HBV-specific CD4 and CD8 T cells, and a further stop NUC phase is ongoing for this study [64]. Elebsiran + BRII-179 (therapeutic vaccine) is another combination that has entered phase II trials, with combination therapy inducing mean HBsAg reduction by 1.7 log IU/ml at EOT [65].

### Combination of RNA silencers with T cell modulators

The combination of xalnesiran with RO7191863 (a PD-L1 locked nucleic acid [LNA]) has been studied in NUC-treated patients. A concurrent regimen (xalnesiran every 4 weeks for 24 weeks + RO7191863 weekly from weeks 13–24) and sequential regimen (xalnesiran every 4 weeks for 24 weeks + RO7191863 weekly from weeks 25–36) were studied, and both regimens led to comparable HBsAg suppression. At EOT, 6.1% of patients (2 out of 33) in the concurrent arm and 12.9% (4 out of 31) in the sequential arm achieved HBsAg seroclearance. Only 2 patients (6.5%) from the sequential arm had sustained HBsAg seroclearance at 24 weeks post-EOT [66].

The OCTOPUS-1 trial is a phase II trial studying the combination of daplusiran/tomligisiran + nivolumab in NUC-treated patients. At EOT, the combination regimen led to mean HBsAg reduction by 2.01–2.10 log IU/ml. The HBsAg levels remained at 1.70–1.85 log IU/ml below baseline at 24 weeks post-EOT, with 72–84% of patients remaining at HBsAg < 100 IU/ml at 24 weeks post-EOT [67].

The combination of elebsiran + nivolumab + selgantolimod (TLR8 agonist) is currently studied in a phase II trial. The triple drug regimen led to HBsAg loss at 24 weeks post-EOT in 2.5% of patients (1 out of 40) who were not on NUCs at baseline. In contrast, none of the non-NUC patients on nivolumab + selgantolimod achieved HBsAg loss. Off-treatment rebound in HBsAg was noted after completion of elebsiran [68].

### Combination of RNA silencers with monoclonal antibodies

The phase II MARCH trial studied the combination of elebsiran + tobevibart (monoclonal antibodies), with or without Peg-IFNα for up to 48 weeks. Among patients with baseline HBsAg < 1000 IU/ml, 38.9% (7 out of 18 patients) on elebsiran + tobevibart and 45.5% (5 out of 11 patients) on elebsiran + tobevibart + Peg-IFNα achieved HBsAg seroclearance at EOT. No patients on tobevibart monotherapy achieved HBsAg seroclearance. The triple combination regimen enhanced the sustainability of HBsAg suppression after EOT [69].

### Evidence of immune dysfunction recovery

In some of the aforementioned trials, recovery of host immune function alongside HBsAg reduction was evidenced by upregulation of soluble inflammatory markers such as IL-2 and IL-6 (e.g., in imdusiran + PEG-IFNα [60]; and in nivolumab + selgantolimod or ledipasvir/sofosbuvir [55]), anti-HBs seroconversion with or without rising titers (e.g., in elebsiran + tobevibart ± PEG-IFNα [69]), and HBV-specific T-cell responses (e.g., in VIR-2218 + selgantolimod + nivolumab [68]). However, these responses were not always associated with HBsAg seroclearance. The association between treatment-induced immune upregulation and functional cure remains unclear, and further research in this area is warranted.

## Discussion

**Fig. 3:**
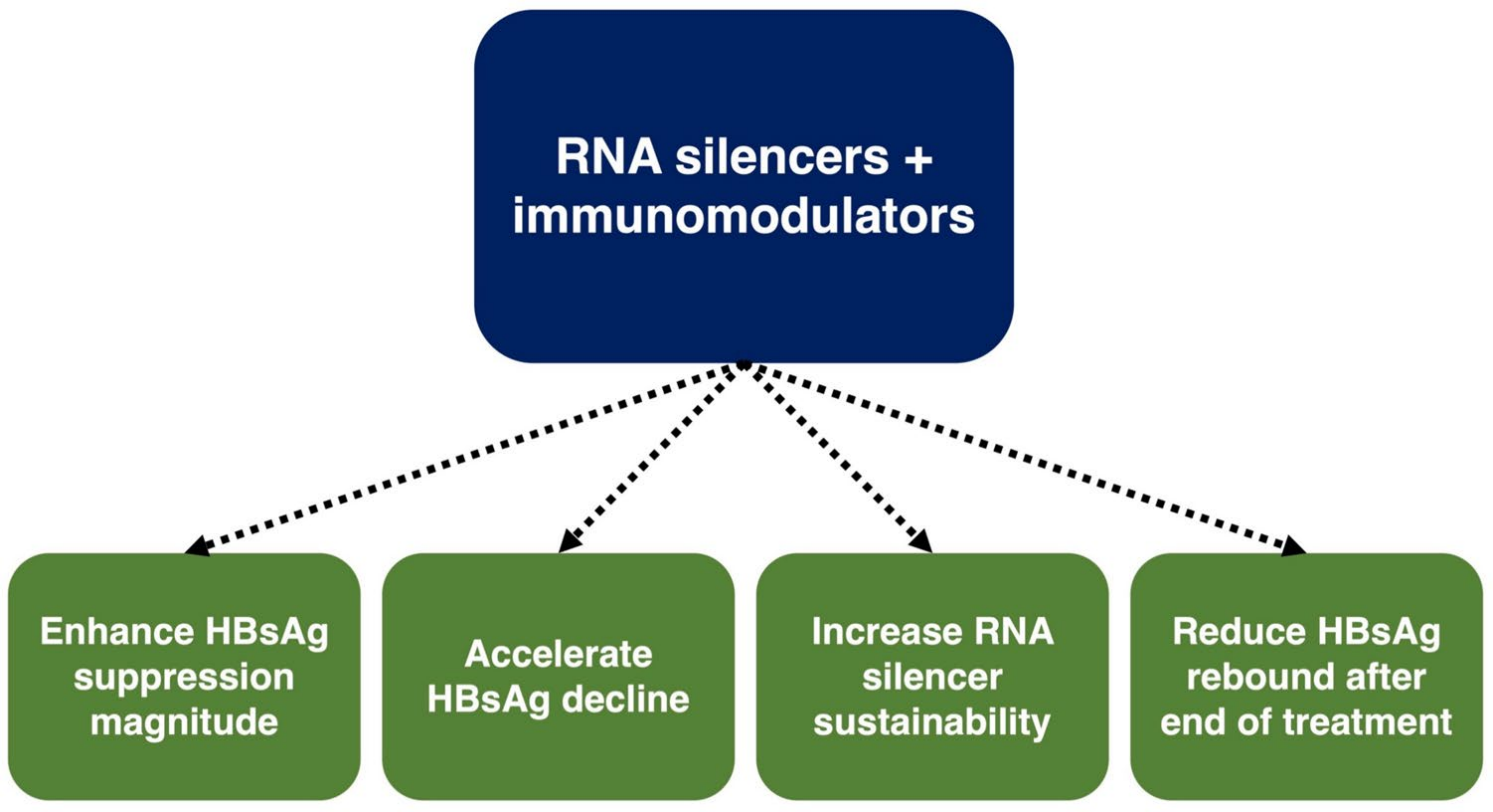
Effects from combining RNA silencers with immunomodulators

This article summarized the numerous completed and ongoing trials on novel combination strategies. The study results are promising, and functional cure is now an attainable target with combination treatment. RNA silencers have been the backbone of most combination strategies, and the addition of immunomodulator adjuncts yields synergistic antiviral effects. Immunomodulators enhance the magnitude of HBsAg suppression [59], accelerate HBsAg decline [61], sustain RNA silencer effects [62, 69], and reduce post-EOT HBsAg rebound [56] (Fig. 3). The combination of immune checkpoint inhibitors with another immunomodulator is also gaining popularity in clinical trials for HBV infection.

Despite the encouraging results, the current trials highlighted that combination strategies require complex design, and patients should not be blindly prescribed a cocktail of drugs. The trials on xalnesiran + RO7191863 [66] and VTP-300 + nivolumab [38] highlighted that the timing of drug administration may influence outcomes, as treatment effects can be negated by drug interactions. Conversely, appropriate design of treatment sequences can leverage the immune reconstitution effects of RNA silencers to potentiate immunomodulator effects [18, 70]. Aside from treatment sequence, the optimal drug dosage and treatment durations also warrant further research. The evidence from current trials will enable optimization of combination treatment strategies, and subsequent validation in larger phase III trials is required.

Lower pre-treatment HBsAg consistently predicted HBsAg seroclearance in monotherapy with novel agents [20, 38, 44], and this association was enhanced in combination treatment trials [56, 57, 59]. Yet aside from baseline HBsAg, our understanding on treatment response predictors remains limited. Other virological markers such as longitudinal qHBsAg kinetics [71–73], HBV RNA [74], and hepatitis B core related antigen (HBcrAg) [75] can predict HBsAg seroclearance in CHB patients, but have not been specifically studied in novel therapy. The combined use of different HBV biomarkers for predicting HBsAg seroclearance in novel therapies should be explored [76]. With the importance of host immunity in CHB, immune markers may also predict HBsAg seroclearance [77–79]. Both peripheral blood and intrahepatic immunology markers have been studied as predictors of treatment responses to novel therapies, although these studies remain exploratory in nature [80, 81]. Identification of response predictors will influence patient selection for combination treatment, and is hence an important area of research.

**Fig. 4:**
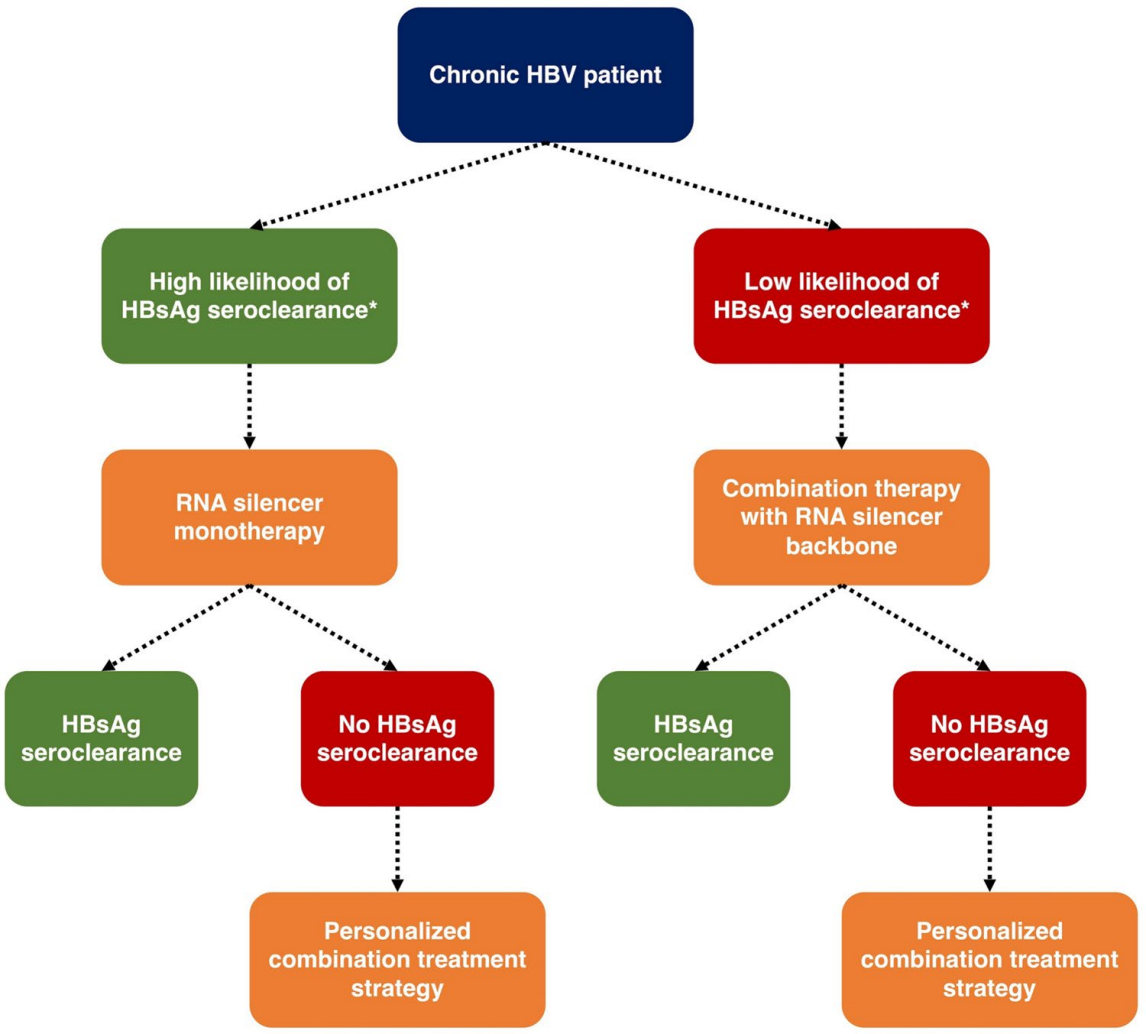
Proposed treatment algorithm with novel therapies. *Further studies are required to assess the predictors of HBsAg seroclearance. Virological factors (including qHBsAg, HBcrAg and HBV RNA) and immunologic factors (including cytokine and immune cell profiles) will both have important roles for prediction and personalization of treatment strategies

The future of CHB treatment will likely involve personalized design of drug regimens. In patients with high likelihood of HBsAg seroclearance (i.e., low baseline HBsAg plus other favorable immunology markers), a single course of ASO or siRNA monotherapy may already be sufficient to induce HBsAg seroclearance. Conversely in patients with low likelihood of treatment response (i.e., high baseline HBsAg plus unfavorable immunology markers), the use of RNA silencers plus immunomodulator adjuncts may be required. The type, number, and administration timing (i.e., concomitantly or sequential) of immunomodulator adjuncts should be further deliberated on an individual patient basis. Our proposed treatment algorithm is summarized in Fig. 4.

Patient tolerability is another important consideration in combination treatment, as patients will inevitably be exposed to adverse effects of 2 or more drugs. RNA silencers are generally safe and well tolerated, although treatment-emergent adverse events such as injection site reactions and flu-like symptoms are common [19]. Peg-IFNα—a drug notorious for its poor tolerability, also has a prominent role in combination strategies, and will certainly influence patient tolerability. Taking the xalnesiran + Peg-IFNα or ruzotolimod trial as an example, treatment-emergent adverse events occurred in 100% of patients in the xalnesiran + Peg-IFNα arm and in 76% of patients in the xalnesiran + ruzotolimod arm [57]. Similarly in the elebsiran + Peg-IFNα trial, treatment-emergent adverse events were common in the cohorts that received the largest number of Peg-IFNα doses (83% in cohort 4 [6 doses of elebsiran + up to 48 doses of Peg-IFNα]; 100% in cohort 5 [up to 13 doses of elebsiran + up to 44 doses of Peg-IFNα]) [58]. Careful patient counseling and close monitoring will be necessary in combination treatment. A limitation of current clinical trials is the inclusion of highly selected patient cohorts. Trials generally exclude patients with cirrhosis or other medical comorbidities. Elderly patients or patients with relatively high qHBsAg (> 3000 IU/ml) were also excluded. The accumulation of real-world safety data will be necessary prior to widespread application of novel combination therapies.

To conclude, novel combination strategies for CHB are emerging. RNA silencers + immunomodulators have demonstrated promising results, and functional cure is now attainable. With further development, novel combination strategies have the potential to transform future CHB management.
